# Industrial Air Pollution and Respiratory Health Status among Residents in an Industrial Area in Central Italy

**DOI:** 10.3390/ijerph17113795

**Published:** 2020-05-27

**Authors:** Giulia Paolocci, Lisa Bauleo, Ilenia Folletti, Nicola Murgia, Giacomo Muzi, Carla Ancona

**Affiliations:** 1 Section of Occupational Medicine, Respiratory Diseases and Toxicology, University of Perugia, 129 Perugia, Italy; giulia.paolocci@unipg.it (G.P.); nicola.murgia@unipg.it (N.M.); giacomo.muzi@unipg.it (G.M.); 2 Department of Epidemiology, Lazio Regional Health Service, ASL Roma 1, 147 Rome, Italy; l.bauleo@deplazio.it (L.B.); c.ancona@deplazio.it (C.A.)

**Keywords:** air pollution, lung function reduction, respiratory symptoms

## Abstract

The area of Civitavecchia (Lazio region, Central Italy) has been a reason of concern in the past because of environmental air contamination. The aim of this study was to evaluate the association between air pollution from different sources and respiratory symptoms and lung function in the population. A sample of 1177 residents underwent medical examination and lung function tests. Information on individual characteristics, histories of exposure and medical history were collected through a validated questionnaire. Long-term exposure to industrial, harbour, biomass combustion emissions (PM_10_) and urban traffic (NO_x_) at residential address was assessed using a Lagrangian dispersion model. The associations between exposure and wheezing and dyspnea were assessed using logistic regression models, while modified Poisson regression models were used to evaluate cough with phlegm. Relationships between exposure and lung function were analysed using linear mixed-effects models and cross-correlation. PM10 emissions from the harbour were associated with lower lung function parameters (FEV1: β = –0.12, 95% CI –0.21 –0.03; *p* = 0.02; FEV1/FVC: β = –1.67, (–3.10 –0.23); *p* = 0.02. This association was observed also in healthy subjects, but not in females. We found, even if at low exposure level, an effect of environmental PM_10_ exposure from harbour on lung function.

## 1. Introduction

Air pollution (AP) is the most relevant environmental cause of disease and premature death in the world today. Diseases caused by AP were responsible for an estimated 9 million premature deaths in 2015, 16% of all deaths worldwide [1]. AP is a toxic mixture of gases and particulate matter (PM) composed of organic chemicals (e.g., polycyclic aromatic hydrocarbons, PAH), metals (e.g., iron, nickel), gases (e.g., ozone), biological agents (e.g., plant pollen, endotoxins, bacteria) and minerals (e.g., quartz, asbestos); variations in compositions are related in each location to meteorological conditions and differences in human activities over time [2]. Air pollutants can cause acute inflammation; human, animal and in vitro experimental studies demonstrated an increased recruitment and activation of inflammatory cells and mediators, as well as activation of intracellular oxidative stress via the generation of free radicals and depletion of protective antioxidants and their enzymes [3]. Respiratory symptoms such as wheezing, cough and shortness of breath are common in respiratory diseases characterized by airway inflammation such as COPD (chronic obstructive pulmonary disease) and asthma [4]. Associations between particulate matter exposure and respiratory health outcomes such as asthma, COPD and lung function decline have been established [5,6]. In a recent study, wheeze, attacks of shortness of breath, bronchitis symptoms and lung function impairment were significantly higher in subjects living within 100 m from the main road who had the highest self-reported exposure to traffic [7]. A longitudinal study among 224 individuals from a Chinese community population founded a significant dose–response relationship of short- and long-term personal PM_2.5_-bound polycyclic aromatic hydrocarbons (PAHs) exposures with lung function reduction [8].

The area of Civitavecchia, located along the northern coast of Rome (Central Italy), has been a reason of concern for several years because of various sources of environmental pollutions, including a large harbour, a cement factory, and three big thermoelectric plants, that may potentially affect the residential communities. In addition, air quality in the area is greatly affected by urban traffic and biomass combustion for civil heating. Previous studies carried out in this area have shown high risks for respiratory diseases and lung cancer among both workers [9,10,11] and residents [12,13]; results of a residential cohort study show that industrial pollution in this area was associated with cause-specific mortality of residents when adjusting for both occupational exposure and socioeconomic status [14]. Air pollution—in particular particulate matter, nitrogen dioxide and ozone—can exert its effects on health after acute (short-term) and chronic (long-term) exposures. Long-term exposures are associated with decreased survival and several noncommunicable diseases, including cardiorespiratory conditions and lung cancer. In Europe, the ESCAPE project (European Study of Cohorts for Air Pollution Effects—www.escapeproject.eu) studied the chronic effects of air pollution in the cohorts of adult subjects. The results of ESCAPE show an association between chronic exposure to air pollutants and natural mortality, cardiovascular events, lung, brain, breast and digestive tract cancer. However, some aspects still need to be clarified, for example, the effect of air pollution on respiratory function, especially from pollution sources other than road traffic, such as industrial sources, civil heating and cruise ships

The aim of this study was to evaluate the association between air pollution from industrial plants, harbour, traffic and biomass heating and respiratory symptoms and lung function in the population living in the area.

## 2. Materials and Methods

### 2.1. Industrial Sources

Three thermoelectric power plants are located in the study area: Fiumaretta, Tor Valdaliga South (TVS) and Tor Valdaliga North (TVN). TVN, located about 5 km from the town centre, was the only one active during the study period; it has been active since 1984, producing 2640 MW of power, and it was converted into a coal-fired power plant in 2010. Emissions from the power plants include particulate matter (PM), nitrogen oxides (NOx) and sulphur dioxide (SO_2_). Each year the TVN plant is authorised to emit 2100 tonnes of SO_2_, 3450 tonnes of NOx, 160 tonnes of dust, 2000 tonnes of CO and 195 tonnes of ammonia (source Dec.Min.114 of 05.04.2013). In addition, several other micro pollutants, including various metals such as arsenic, mercury, vanadium, nickel, cadmium, and chromium, are emitted. The flue gases are dispersed into the atmosphere through three metal tubes with similar characteristics: flue gas outlet temperature, 110 °C; flue gas outlet velocity, 16 m/s; chimney internal diameter, 5.7 m. The three pipes are located inside a 250 m high chimney.

The Civitavecchia harbour (241,000 m^2^, 26 operational piers, 23 berths for up to 100 m yachts) has been traditionally used for ferry traffic, merchant ships and tankers. The improvement of docks and structures for passengers over the years allowed for an extraordinary increase of cruise ships (from 50 ships in 1996 to 950 in 2013) and ferries (1500 yearly), with an annual average number of passengers reaching up to 4 million in recent years. 

Air quality in the area is also affected by urban traffic. In 2012, about 84,000 vehicles per day (65,000 cars; 6000 trucks; 13,000 motorcycles) have been estimated as moving in the area, with a large component of vehicles in transit due to the presence of the harbour. During the summer, the traffic increase is of about 10,000 vehicles per day (700 heavy vehicles).

### 2.2. Study Population

Among the residents living in the study area in 1996, a random sample of 2000 subjects (35–69 years) was selected from municipality registers and invited to participate in the ABC (Ambiente Biomonitoraggio Civitavecchia) human biomonitoring study in the period 2013–2016. For each participant, we have at least 17 years of duration of residence [15]. Study participants received a letter of invitation to participate in the study. A total of 1790 residents were contacted by telephone, of which 34% refused to participate. A number of 1177 persons (66% of those contacted by telephone) agreed to participate. A total of 1141 medical examinations were available. There was no significant difference in term of gender, age and municipality of residence among people with medical examination versus people without. Their residence addresses were geocoded using GIS (Geographic Information System) techniques. A total of 1177 subjects (66% of those contacted) agreed to participate and underwent medical interview. A face-to-face questionnaire was administered to collect information on individual characteristics, consumption of local food, occupational history, lifestyle and medical history. People were asked about the presence of current respiratory symptoms (including shortness of breath, wheezing and cough with phlegm in the last 12 months) (no, yes) and about pharmacological therapies at home (no, yes), and they were asked to provide a written consent for the medical examination and spirometry. The items were largely based on the questionnaire of the Italian Studies on Respiratory Disorders in Children (SIDRIA) [16]. 

All the subjects underwent a general medical examination, lung function tests and urine collection for cotinine measurement.

From the questionnaire items, we identified sociodemographic (occupational status, educational level and body mass index (BMI)) and behavioural risk factors (smoking status) to be included as a priori potential confounders and to define susceptible subgroups. The physician diagnosis of asthma, emphysema, chronic bronchitis and COPD, reported in the ABC questionnaire, was also considered. Healthy subjects were those who did not report physician diagnosis of asthma, emphysema, chronic bronchitis and COPD in the ABC questionnaire. We considered exposure to vapours, dusts, gases and fumes (VGDF) occurring the week before the ABC examination to evaluate the role of occupational exposure.

### 2.3. Exposure Assessment

The residential cohort study recently conducted in the area to assess the effects of industrial emissions on the health of residents [14] has made available the ground-level footprints of the pollutants chosen as tracers for each source under study (PM_10_ for the TVN plant, ship emissions and civil heating, NOx for traffic). 

In summary, a modelling simulation domain (50 × 50 km) has been defined that includes all the municipalities around Civitavecchia. The annual average concentration of ground fallout has been estimated for each 500 × 500 m cell. The dispersion model used was the Lagrangian nonstationary particle model SPRAY (ARIANET Srl. SPRAY. www.aria-net.it/front/IT/codici/files/10.pdf). The PM_10_ ground footprint for TVN was obtained by considering the emission quantities authorized in 2012, their temporal modulation and the emission mode (release height, smoke speed and relative temperature).

As for the harbour, in 2012, information about the number of ships and ferries docked to each port quay and the stay at the port (arrival date, arrival time, departure date, departure time, number of the warehouse), time and path of entry manoeuvre, parking and manoeuvre exit of each ship was used to estimate the PM_10_ concentrations due to ship emissions. 

Outdoor concentrations of NO_x_ at the residential address were estimated as tracers of urban traffic pollution. Data for dispersion model simulation were estimated from two road traffic measurement campaigns (one in winter and one in summer 2014) in the area. 

Finally, as biomass combustion is widely used in the area for heating purposes, data about number of chimneys and wood stoves were available from a previous survey conducted in the area [15].

Specifically, this estimate was made within each 500 × 500 m cell based on the resident population. Each cell was considered as an area source, and the emissions were calculated using the emission factors estimated by Ozgen et al. [17] and only for the months from November to March.

### 2.4. Clinical Examination and Lung Function Assessment

For each subject, measurements of body weight (Kg) and standing height (cm) were performed. A trained physician specialized in respiratory medicine evaluated lung function during the ABC clinical visits. Lung function was measured by spirometry based on standard operating procedures using COSMED spirometer device system according to ATS/ERS recommendations [18]. The same device was used throughout the study. At least three successful forced expiratory manoeuvres, using a nose clip, out of a maximum of eight trials, were required for each subject. The operators accepted only forced expirations which met the criteria recommended by the ATS/ERS [18]. The following spirometric indices were calculated from each expiration: forced vital capacity (FVC), forced expiratory volume in one second (FEV1) and forced expiratory flow at 25% to 75% (FEF25–75). Urinary cotinine levels were collected to assess exposure to cigarette smoke [19].

### 2.5. Statistical Analysis

PM_10_ (thermoelectric plant, harbour and biomass combustion) and NO_x_ (traffic) concentrations were modelled as a fixed continuous variable using the annual mean exposure estimated at the resident’s home address. Because of the varying magnitude of the exposure indicator, the linear association was estimated for an increment equal to the difference between the 95th and the 5th percentile of the distribution of each pollutant.

Descriptive analyses were conducted, and for continuous variables means and standard deviations (SD) were calculated. The associations between the exposure (as continuous variables) and the dichotomous outcomes (wheezing and dyspnea) were assessed using logistic regression (odds ratio, ORs and corresponding 95% CIs), while modified Poisson regression models [20] (relative risks, RRs and corresponding 95% CIs) were used to evaluate the relationship among exposure variables and cough with phlegm because of the relatively high prevalence of the outcomes in the study sample. Finally, relationships between exposure and lung function were analysed using linear mixed effects models and cross-correlation. We included in the analysis the smoking status derived from the questionnaire that allowed us to distinguish whether the person was an active smoker, non-smoker or ex-smoker. The additional information on the urinary cotinine allowed us to take into account possible exposure to second-hand smoke. A sensitivity analysis on healthy subjects (who had negative answer to the question “Have you ever had a physician diagnosis of asthma or/and COPD or/and emphysema or/and chronic bronchitis?”) and by gender was performed. SAS (SAS Institute Inc, North Carolina, NC, USA) and STATA ver.12 (Stata Corp, Texas, TX, USA) software programs were used for the statistical analyses. 

## 3. Results

**Figure 1:**
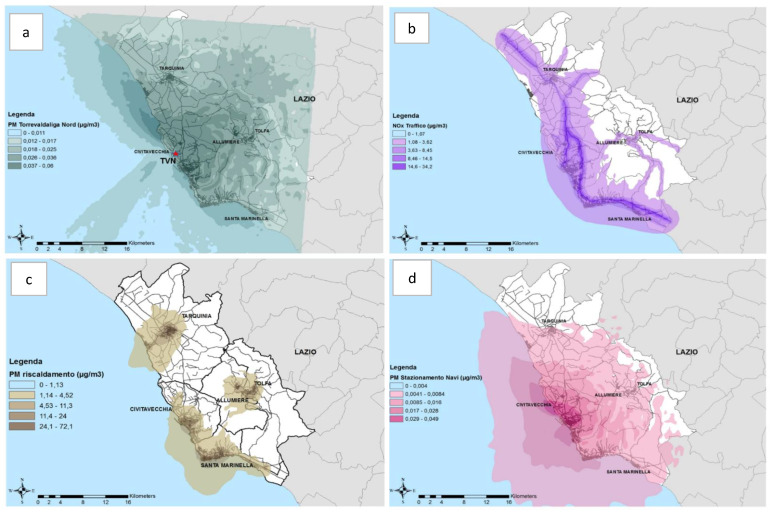
Results of the dispersion model from PM_10_ from thermoelectric power plant (panel **a**), nitrogen oxides (NOx) from traffic (panel **b**), PM_10_ from biomass burning (panel **c**) and PM_10_ from harbour (panel **d**).

We analysed 1141 (57.8% females) ABC study participants who completed the medical examination, answered the questionnaire and performed lung function assessment. Figure 1 shows PM_10_ from TVN thermoelectric plant concentrations (a), urban traffic NO_x_ (b), shipping emissions PM_10_ (c) and biomass heating PM_10_ (d) [21]. Correlations among pollutant concentrations estimated were generally low, with the exception of PM_10_ from TVN and PM_10_ from harbour due to the geographical proximity (r = 0.62).

Annual average exposure levels of the study sample (mean, SD) to PM_10_ of industrial origin (TVN) were 0.03 µg/m^3^ (0.008), 6.32 µg/m^3^ (4.09) for NO_x_ (urban traffic), 0.02 µg/m^3^ (0.01) for PM_10_ (shipping emissions) and 9.59 µg/m^3^ (9.88) for PM_10_ (biomass combustion from civil heating).

The individual characteristics of the sample according to exposure categories, PM_10_ from TVN, harbour and biomass combustion and NO_x_ from traffic, are described in Table 1 (low level of exposure, <5th; medium level of exposure, 5th–95th; high level of exposure, >95th percentile cut-offs). The distribution of gender was rather similar over the exposure categories. The mean age was 53.9 years (SD, 10) in men and 53.5 (SD, 9.6) in women. People living in the area with higher concentrations of PM_10_ or NO_x_ were more likely to be younger and with a lower educational level compared with people living in areas with lower exposure. Almost 50% of the subjects were employed, and 24% of them referred to have been exposed to vapours, dusts, gases and fumes (VGDF) in the week before the clinical examination. Of the subjects, 24% were current smokers.

As for the body mass index (BMI, calculated as the ratio of weight in kilograms and the square of height in meters), 42% of the study group had a BMI greater or equal than 25, the value defined by the WHO as a cut-off point for overweight. A total of 8.5% of the subjects reported physician-diagnosed asthma; 2% of the subjects declared emphysema and 4.5% chronic bronchitis. Only two subjects had a physician-diagnosed COPD. 

Subjects reported a higher prevalence of wheezing with increasing exposure to industrial PM_10_ (4.1%, 10.4%, 13.6%, respectively), while higher prevalence of cough with phlegm was observed among people more exposed to traffic NOx (46.4%) (Table 2). Average levels of lung function were FEV1, 3.05 L (0.80 SD); FVC, 3.85 L (0.99 SD); FEF25–75, 2.96 L (1.02 SD) and the FEV1/FVC, 79.53% (7.58 SD), with no differences across exposure categories (Table 2). 

Table 3 shows the results of the association of pollutants concentrations with respiratory symptoms. After adjustment for gender, age, body mass index, smoking status, cotinine level, other environmental exposure (PM_10_ from TVN, NO_x_ from traffic, PM_10_ from harbour and PM_10_ from biomass combustion), educational level, occupation, and exposure to VGDF, we found some positive associations (TVN and wheezing, shipping emissions and cough with phlegm, biomass combustion and cough with phlegm), however those associations did not reach statistical significance.

Table 4 shows the results of the association of pollutant concentrations with lung function. No association was found for emissions from TVN, biomass or urban traffic; however, ship emissions were associated with decrease in FEV1 (beta = –0.12; CI 95% –0.21. –0.02), FEV1/FVC (beta = –1.67; CI 95% –3.10. –0.23), and FEF25–75 (beta = –0.12; CI 95% –0.21. –0.02). An exposure greater than 0.04 µg/m^3^ in PM_10_ concentration from the harbour was associated with an FEV1 and FEF25–75 reduction of 12 mL.

The sensitivity analyses conducted on healthy subjects and by gender confirmed a relationship between lung function decline and PM10 exposure from ship emissions similarly, PM_10_ exposure from the harbour had a role in the reduction of FEV1, FEV1/FVC and FEF25–75, mostly in men.

## 4. Discussion

We found associations between PM_10_ concentrations from the ship emissions and a reduction of lung function when demographic characteristics, smoking habits, socioeconomic status and current occupational exposures were taken into account. Furthermore, exposure to PM_10_ from the biomass combustion seems to be correlated to cough with phlegm. The associations found at estimated exposure levels were rather low. PM_10_ confirms its role as respiratory hazard, especially for obstructive lung diseases [22]. These results concur with several previous studies that have reported an association between ambient PM concentrations and increased prevalence of cough [23], even if in children. A large cross-sectional study in Switzerland found an association between increased prevalence of chronic cough and phlegm with PM10 exposure among never-smokers [24]. The European Study of Cohorts for Air Pollution Effects (ESCAPE) meta-analysis of five European cohorts similarly showed an association between PM_10_ and prevalence of chronic phlegm, but not chronic bronchitis, in never-smokers [25]. A French study of elderly adults demonstrated increased prevalence of chronic cough associated with PM_10_ exposure [26]. Furthermore, in the Swiss Cohort Study on Air Pollution and Lung and Heart Diseases in Adults (SALPADIA) cohort, a decline in PM_10_ over time was associated with a reduction in chronic cough and phlegm [27]. In our study, the association between PM_10_ and respiratory symptoms was weaker and not statistically significant, maybe because of the rather low average estimated concentrations to which our sample have been exposed.

Our study may suggest that air pollution, especially PM_10_, can lead to respiratory damage which could bring a reduction in lung function. The design of this study (cross-sectional) does not allow any speculation about the role of long-lasting or short-term air pollution, both could contribute to the lower levels of lung function parameters observed in those more exposed. Recent studies showed significant associations between increased levels of ultrafine particulate matter and decreased lung function, regarding the exposure to traffic emission [28,29] or living in proximity to a steel plant [30].

In this study, the shape of the concentrations of the “estimated” pollutants on the ground was used to rank subjects as more or less exposed according to their residential address. The major limitation of our exposure assessment is related to the lack of a validation study with in situ measurements. Nonetheless, SPRAY is a consolidated model that has been validated using a “conventional” validation framework [31] and its performances and efficiency have been evaluated and validated in multiple real conditions with different orography, size of domain, number of grid cells in the domain, meteorological conditions and emission types [32].

In our study, an exposure to a PM_10_ concentrations over 0.04 µg/m^3^ from the harbour was significantly associated with a 12 mL lower FEV1 and FEF25–75, comparable to a study by Luc Int Panis et al. published recently [33]. Differently from the results of the Framingham Heart study cohort [5], we found an association between air pollution and the FEV1/FVC ratio with a reduction of 1.67% of FEV1/FVC ratio in subjects more exposed to PM_10_ from harbour.

Furthermore, our data on FEF25–75, especially those obtained in healthy individuals, where this parameter may be a rough proxy of small airways functionality [34], suggest that pathological and functional changes due to air pollution can occur in peripheral airways too, similarly to the effects of cigarette smoking found in other studies [35]. Our results confirmed the hypothesis of possible health effects by ship emissions; a recent study conducted by Barregard et al. showed that environmental policy development on air pollution from shipping emissions in the Baltic Sea can result in a decrease in health impacts [36]. Our data suggest that men may be more susceptible to the effects of air pollution than women, an observation which is not shared by previous studies [37,38], except, in our knowledge, a recent study by Doiron et al. where in males lower lung function was associated with increased air pollution exposure [39].

It is important to emphasize that the association that we found between PM_10_ and lung function reduction occurred at estimated concentrations of pollutants far below the levels of background pollution (for example, in the city of Rome in 2011 the concentration of PM10 was 25 µg/m^3^ and NO_2_ was 40 µg/m^3^ in the urban zone) [40] and also lower than limits suggested by WHO (20 µg/m^3^ for annual average PM_10_ and 40 µg/m^3^ for annual average NO_2_) [41].

The results for this apparently healthy group of adults are novel because few studies have looked at healthy adults. The strength of this work is the standardized questionnaire, cigarette smoking exposure evaluated by cotinine and the environmental exposure assessment with a validated model of dispersion. Our results were adjusted for several confounders: age, socioeconomic position, and variables related to the environmental and occupational context that might otherwise have confounded or modified our results. However, no data were available on all detailed occupational activities in the past. The lack of such information may have biased the results because long-lasting occupational exposure could affect lung function significantly [42], and this is a limitation in our study because we assessed occupational exposure only for one week before ABC examination. Pollutant levels estimated only at the baseline addresses may not adequately represent exposure, because people do not spend all their time at home, and we have not detailed information regarding daily activity.

This is one of the few available studies that analysed and drew attention to the effect on respiratory health of low-dose exposures to environmental air pollutants in an industrial area in Italy. Results related to the harbour support an intervention to reduce ship emissions in the Civitavecchia area. Knowledge of the respiratory health effects of industrial air pollution would offer a valuable rationale for policymakers to implement community-based intervention approaches to reduce personal and population exposure. In the treatment of respiratory diseases, the most effective environmental remediation strategies are multifaceted interventions. However, more studies about the role of environmental remediation need to be conducted in adults compared with children [43]. Reducing the impact of air pollution on respiratory health will require both public policy and the actions of individual patients. Public health experts and campaigners have consistently pointed to the discrepancy between EU air pollution standards and WHO recommendations as an area in need of urgent action. The average annual limit of particulate matter with a diameter less than 2.5 µm set by the EU in 2008 was 25 µg/m^3^, despite WHO recommending a limit of 10 µg/m^3^ in 2005 based on the evidence of detrimental health effects available at the time. Evidence generated since then, summarized in a more recent WHO report, add support to these guideline measures [44].

## 5. Conclusions

In conclusion, this study demonstrates that air pollution may have a negative impact on respiratory status of an healthy adult population residents nearby the Civitavecchia harbour. This association was found at estimated exposure levels rather low. Harbour air pollution can affect both large and small airways. A higher prevalence of wheezing was reported in increasing exposure to industrial PM10 and higher prevalence of cough with phlegm in exposure to traffic NOx. Interventions to reduce ship emissions, industrial PM10 and traffic NOx are recommended for public respiratory health prevention.

## Figures and Tables

**Table 1:** Descriptive individual characteristics of the sample according to exposure categories (low exposure, <5th; medium exposure, 5th–95th and high exposure, >95th percentile cut-offs).

		PM_10_ From Thermoelectric Power Plant			NOx From Urban Traffic			PM_10_ From Shipping Emissions			PM_10_ From Biomass Combustion		
		<5th (<0.02)	5°–95° (0.02–0.04)	>95th (>0.04)	<5th (1.89)	5°–95° (1.89–16.58)	>95th (>16.58)	<5th (<0.00)	5°–95° (0.00–0.04)	>95th (>0.04)	<5th (<1.65)	5°–95° (1.65–33.98)	>95th (>33.98)
***Total sample (%)***	***1141***	***49 (4)***	***1026 (90)***	***66 (6)***	***57 (5)***	***1015 (89)***	***69 (6)***	***46 (4)***	***1019 (89)***	***76 (7)***	***57 (5)***	***1023 (90)***	***61 (5)***
**Gender, %**	***Males***	34.7	42.9	39.4	38.6	42.9	37.7	41.3	42.7	38.2	49.1	42.0	41.0
	***Females***	65.3	57.1	60.6	61.4	57.1	62.3	58.7	57.3	61.8	50.9	58.0	59.0
**Age class, %**	***35–44***	22.4	22.4	21.2	17.5	22.3	27.5	17.4	22.3	26.3	21.1	22.7	18.0
	***45–54***	36.7	30.3	27.3	33.3	30.0	33.3	21.7	30.9	28.9	29.8	29.9	39.3
	***55–64***	30.6	29.4	36.4	33.3	30.0	26.1	43.5	29.1	31.6	38.6	29.7	24.6
	***>= 65***	10.2	17.8	15.2	15.8	17.7	13.0	17.4	17.7	13.2	10.5	17.7	18.0
**Educational levels, %**	***None/elementary***	8.2	9.3	4.5	14.0	8.6	10.1	15.2	8.5	10.5	7.0	9.3	4.9
	***Lower secondary school***	34.7	30.4	24.2	35.1	30.0	30.4	34.8	30.2	27.6	33.3	29.8	34.4
	***Upper secondary school***	40.8	51.1	60.6	43.9	51.3	55.1	41.3	51.6	51.3	52.6	51.2	49.2
	***Graduate/higher qualification***	16.3	9.3	10.6	7.0	10.1	4.3	8.7	9.6	10.5	7.0	9.7	11.5
**Occupational status, %**	***Employed***	55.1	53.5	48.5	52.6	53.3	53.6	45.7	54.0	48.7	63.2	53.0	49.2
	***Unemployed***	4.1	5.4	1.5	1.8	5.3	4.3	4.3	5.0	6.6	3.5	5.0	8.2
	***Housewife***	32.7	19.9	27.3	28.1	20.4	21.7	30.4	20.2	23.7	17.5	20.7	26.2
	***Retired/invalid***	8.2	21.2	22.7	17.5	21.0	20.3	19.6	20.8	21.1	15.8	21.3	16.4
**Smoking status, %**	***Current smoker***	30.6	24.0	25.8	17.5	25.1	18.8	17.4	24.4	27.6	14.0	25.4	16.4
	***Never-smoker***	46.9	39.2	39.4	56.1	38.3	43.5	41.3	39.7	35.5	40.4	39.6	37.7
	***Ex-smoker***	22.4	36.8	34.8	26.3	36.6	37.7	41.3	35.8	36.8	45.6	35.0	45.9
**Body Mass Index, %**	***Underweight (<18.49)***	0.0	1.0	0.0	1.8	0.8	1.4	0.0	0.9	1.3	0.0	1.0	0.0
	***Normal weight (18.5–24.99)***	26.5	32.8	28.8	31.6	32.2	34.8	21.7	32.7	34.2	21.1	32.7	36.1
	***Overweight (25–29.99)***	57.1	41.9	36.4	38.6	42.2	46.4	52.2	41.5	46.1	42.1	42.2	42.6
	***Obese (30–34.99)***	12.2	17.3	25.8	22.8	17.6	11.6	19.6	17.8	13.2	29.8	17.1	13.1
	***Severely obese (>34)***	4.1	7.0	9.1	5.3	7.2	5.8	6.5	7.2	5.3	7.0	6.9	8.2
Exposure to VGDF *at work, %		12.2	24.5	19.7	28.1	23.3	26.1	23.9	23.7	22.4	36.8	23.5	14.8
**Asthma, %**		4.1	8.6	12.1	5.3	8.7	10.1	6.5	8.3	13.2	10.5	8.0	16.4
**Emphysema, %**		0.0	2.4	0.0	1.8	2.2	2.9	0.0	2.5	0.0	0.0	2.2	3.3
**Chronic Bronchitis, %**		2.0	4.9	1.5	0.0	0.0	0.0	6.5	4.7	1.3	3.5	4.4	8.2
**COPD ^#^, %**		0.0	0.2	0.0	0.0	0.0	0.0	0.0	0.2	0.0	0.0	0.2	0.0

* Vapour, gas, dust and fumes (previous week); # chronic obstructive pulmonary disease.

**Table 2:** Respiratory symptoms and lung function by level of exposure.

		PM_10_ from Thermoelectric Power Plant			NOx from Traffic			PM_10_ from Harbour			PM_10_ from Biomass combustion		
		<5th (<0.02)	5°–95° (0.02–0.04)	>95th (>0.04)	<5th (1.89)	5°–95° (1.89–16.58)	>95th (>16.58)	<5th (<0.00)	5°–95° (0.00–0.04)	>95th (>0.04)	<5th (<1.65)	5°–95° (1.65–33.98)	>95th (>33.98)
***Total Cohort, n***	***1141***	***49***	***1026***	***66***	***57***	***1015***	***69***	***46***	***1019***	***76***	***57***	***1023***	***61***
**Wheezing, %**		4.1	10.4	13.6	14.0	9.9	14.5	8.7	10.5	9.2	8.8	10.5	9.8
**Dyspnea, %**		10.2	10.6	7.6	3.5	10.6	13.0	15.2	10.6	5.3	12.3	10.3	11.5
**Cough with phlegm, %**		46.9	43.3	36.4	38.6	43.1	46.4	47.8	42.4	48.7	47.4	42.6	45.9
**FVC, L (SD)**		3.81 (1.08)	3.85 (0.99)	3.83 (0.93)	3.81 (0.89)	3.85 (0.89)	3.77 (1.02)	3.86 (0.95)	3.84 (1.00)	3.86 (0.88)	4.06 (1.07)	3.83 (0.99)	3.91 (0.97)
**FEV1, L, (SD)**		3.05 (0.84)	3.05 (0.80)	3.03 (0.72)	3.05 (0.72)	3.06 (0.80)	2.96 (0.91)	3.02 (0.70)	3.05 (0.81)	3.06 (0.75)	3.19 (0.78)	3.04 (0.80)	3.10 (0.80)
**FEF25–75, L (SD)**		2.97 (0.96)	2.97 (1.03)	2.85 (0.88)	2.98 (0.80)	2.96 (1.02)	2.93 (1.14)	2.74 (0.78)	2.97 (1.03)	2.93 (1.01)	3.00 (0.83)	2.97 (1.03)	2.82 (0.91)
FEV1/FVC * **(SD)**		80 (4.98)	79.51 (7.79)	79.37 (5.89)	80.00 (7.40)	79.56 (7.21)	78.63 (12.13)	78.65 (5.17)	79.59 (7.70)	79.36 (7.40)	79.21 (6.06)	79.57 (7.72)	79.30 (6.62)

FEV1, forced expiratory volume in 1 s in litres, mean and standard deviation (SD); FVC, forced vital capacity in litres, mean and standard deviation (SD); FEF25–75, forced expiratory flow between 25% and 75% of the FVC in litres, mean and standard deviation (SD); * (FEV1/FVC) × 100, forced expiratory volume in 1 sec to forced vital capacity ratio percent, mean and standard deviation (SD).

**Table 3:** Association between PM10 from thermoelectric power plant, from harbour, from biomass combustion, and NOx from traffic and respiratory symptoms. Adjusted odds ratios (ORs and 95% CI).

	PM_10_ from Thermoelectric Power Plant	NOx from Traffic	PM_10_ from Harbour	PM_10_ from Biomasscombustion
	OR, (95% CI)	OR, (95% CI)	OR, (95% CI)	OR, (95% CI)
**Wheezing ^a^**	1.65, (0.66–4.10)	0.86, (0.37–1.96)	0.59, (0.26–1.29)	0.53, (0.24–1.17)
**Dyspnea ^a^**	0.79, (0.32–1.94)	1.25, (0.59–2.67)	0.82, (0.37–1.81)	0.80, (0.39–1.62)
**Cough with phlegm ^a^**	0.88, (0.66–1.17)	1.08, (0.34–1.38)	1.09, (0.85–1.41)	1.13, (0.92–1.39)

^a^ Adjusted for gender, age, body mass index, smoking status, cotinine level, other environmental exposure, educational level, occupation, exposure to VGDF.

**Table 4:** Association between PM10 from thermoelectric power plant, form harbour, from biomass combustion, and NOx from traffic and lung function. Regression coefficient (β and 95% CI).

	PM_10_ from Thermoelectric Power Plant	NOx from Traffic	PM_10_ from Harbour	PM_10_ from Biomasscombustion
	β ^@^, (95% CI)	β ^@^, (95% CI)	β ^@^, (95% CI)	β ^@^, (95% CI)
**FEV1 ^a^**	0.00, (−0.08. 0.09)	−0.06, (−0.16. 0.03)	−0.12 *, (−0.21. −0.02)	−0.02, (−0.08. 0.03)
**FEV1/FVC ^a^**	−0.25, (−1.55. 0.04)	−0.07, (−1.54. 1.38)	−1.67 *, (−3.10. −0.23)	−0.59, (−1.49. 0.29)
**FEF25–75 ^a^**	0.00, (−0.08. 0.09)	−0.06, (−0.13. 0.03)	−0.12 *, (−0.21. −0.02)	−0.02, (−0.08. 0.03)

^a^ Adjusted for gender, age, height, body mass index, smoking status, cotinine level, other environmental exposure, educational level, occupation, exposure to VGDF; * *p* < 0.02. ^@^ expressed in mL.
